# Supplementary figures and images for: Effects of precise cardio sounds on the success rate of phonocardiography
Source: PLoS One. 2024 Jul 15;19(7):e0305404. doi: 10.1371/journal.pone.0305404 (PMC11249217; doi:10.1371/journal.pone.0305404)

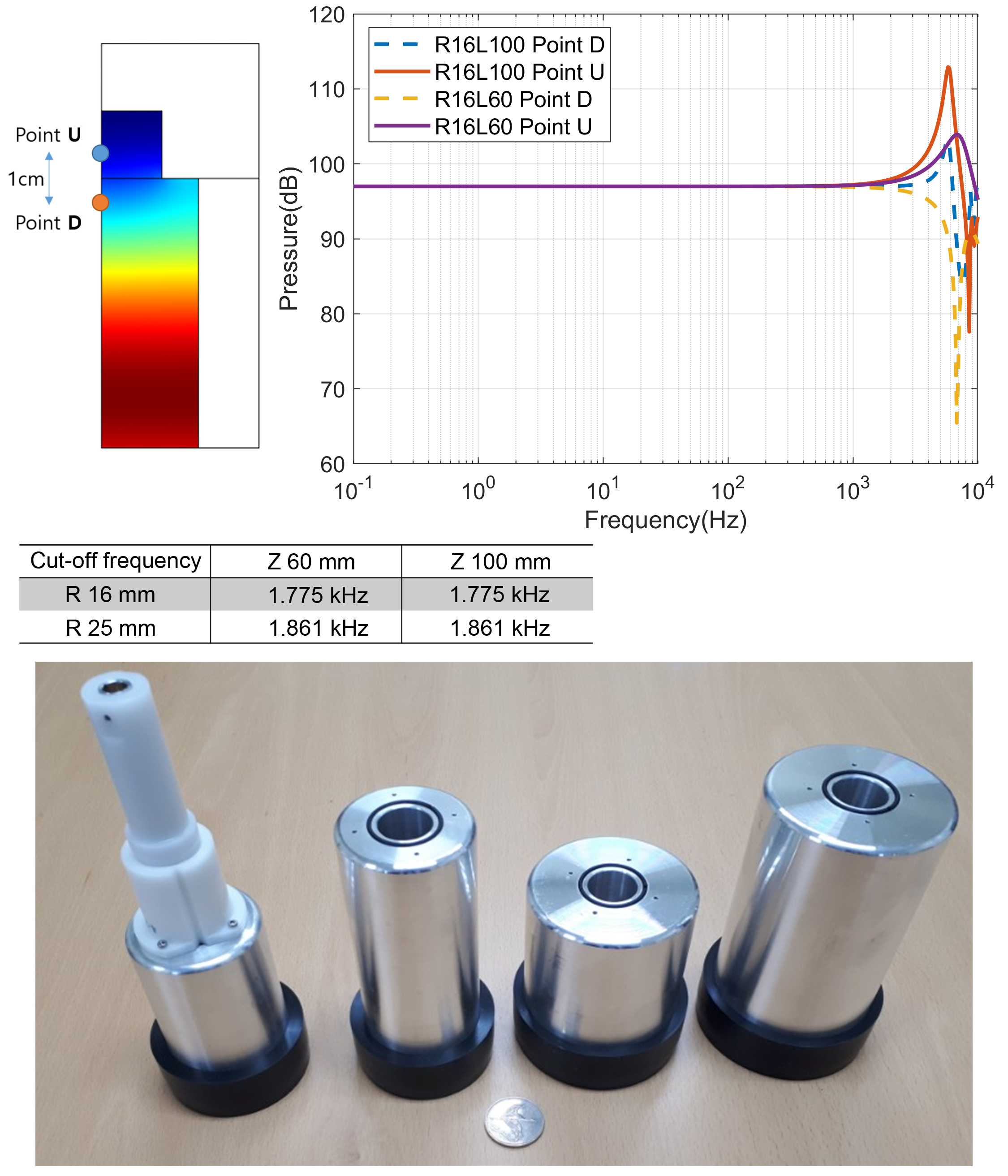

Supplement: S1 Fig — The ARTA acoustic chamber model design method is used to design acoustic adapters that can accurately measure sound pressure in an experimental setting. One important parameter in this design method is the cut-off frequency, which is defined as the frequency at which the sound pressure has a difference of 1 dB per cm displacement in the longitudinal direction. If a detected frequency exceeds the cut-off frequency, it causes a measurement error. Therefore, the designed acoustic adapter must have a cut-off frequency that exceeds the maximum frequency of the sound being measured. In the case of heart sounds, the maximum frequency is known to be 1 kHz. To design the acoustic adapter, finite element modeling was used for calculation according to the design method. A COMSOL model was created, and the sound pressure difference between Point 1, where the microphone is located, and Point 2, which is 1 cm away from Point 1, was calculated. The frequency above which the sound pressure difference exceeds 1 dB becomes the cut-off frequency of the acoustic adapter. By following this design method, geometric conditions that satisfy the required cut-off frequency can be found and designed. This result ensures that the acoustic adapter can accurately measure sound pressure in the experimental setting; this ability is important for increasing the accuracy and effectiveness of diagnostic tools for cardiovascular diseases. (TIF) [file pone.0305404.s001.tif]

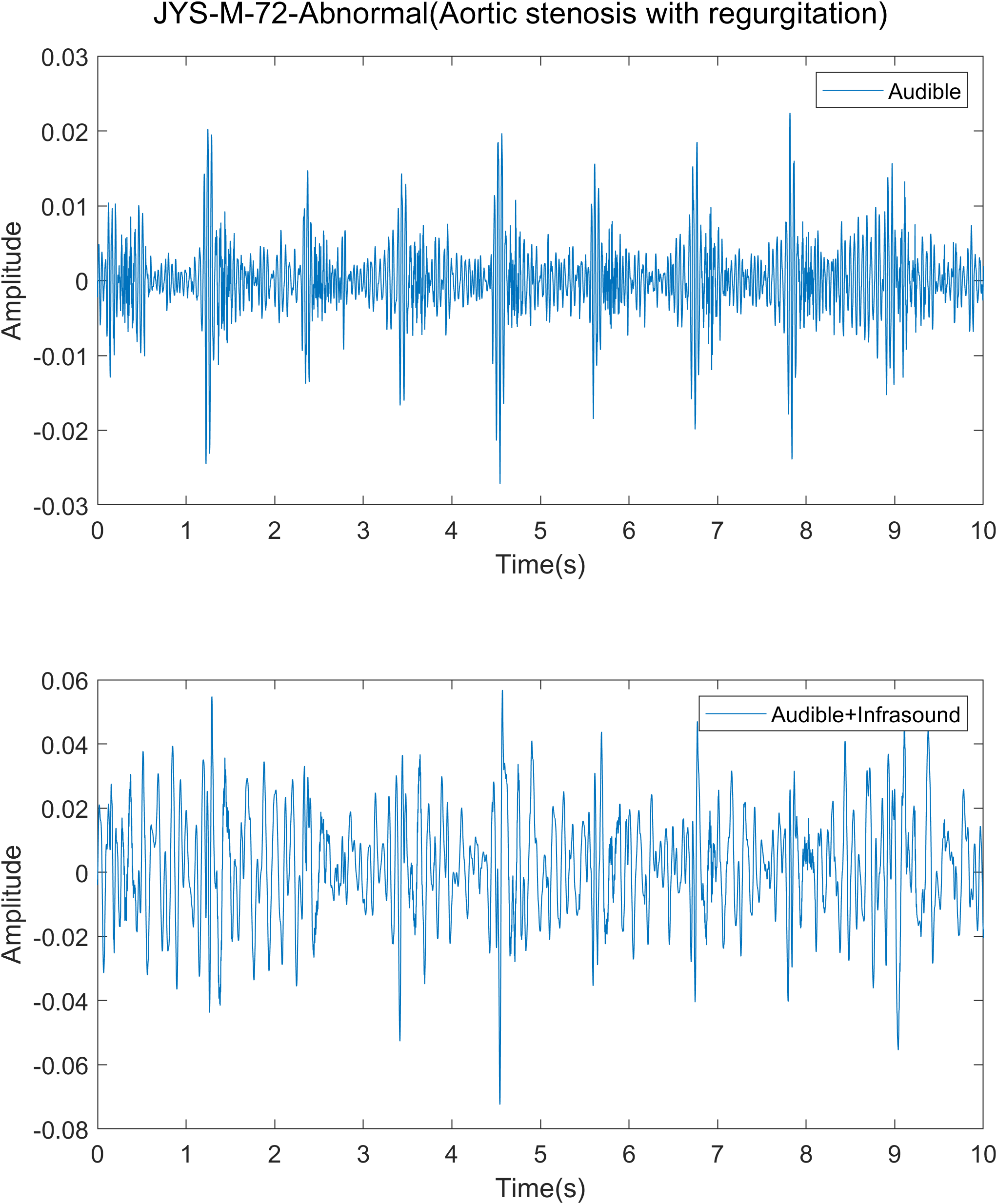

Supplement: S2 Fig — Example of time domain data. (TIF) [file pone.0305404.s002.tif]
