# Supplementary material for: Effects of precise cardio sounds on the success rate of phonocardiography
Source: PLoS One. 2024 Jul 15;19(7):e0305404. doi: 10.1371/journal.pone.0305404 (PMC11249217; doi:10.1371/journal.pone.0305404)
Supplement: Attachment — Submitted filename: Response to reviewers.docx [file pone.0305404.s004.docx]

**Responses to the Associate Editor’s and Reviewer’s Comments**

05 January 2024

Dear reviewers and editorial staffs in *PLOS ONE*

We are profoundly grateful for the insightful critiques and constructive suggestions provided on our manuscript titled **“Effects of precise cardio sounds on the success rate of phonocardiography”**, control number PONE-D-23-23289. These comments have been invaluable in enhancing the scholarly rigor and clarity of our work. In recognition of the meticulous efforts of both reviewers and editors, we have endeavored to refine our manuscript according to the provided suggestions. We believe these enhancements have notably improved the scientific and clinical rigor of our manuscript. We hope that our revised manuscript will be considered and accepted for publication in PLOS ONE. We eagerly anticipate the possibility of our work contributing to the esteemed journal.

In the revised version, we have highlighted all changes for your convenience; these are underlined and colored in blue. This is to ensure that our revisions are easily identifiable and can be efficiently evaluated.

**Reviewer #1:**

***<Review Comments>***

1) **Reviewer’s comment:** To bolster their conclusions in this direction, the authors should design experiments and analyze the dataset more systematically, encouraging the research community to pursue this approach. For instance, the authors need to demonstrate that their 'infrasound' approach outperforms the one based on the widely known PhysioNet dataset. This could involve benchmarking the learning with various models, as it's possible that models other than CNN-Dense and CNN-LSTM may achieve better performance.

**Author’s response**:

If we intend to prove that every diagnosis must be done by use of the true heart sound including its infrasound content, we have to complete the works suggested by Reviewer 1 in the above comments. However, our conclusions are different from the previous statement.

What we claim in the manuscript may be summarized as follows:
1. The infrasound contents should be included in the data for the diagnoses on heart diseases since a lot of infrasound contents are included in the real heart sounds in addition to the audible ones.
2. The true heart sound (audio + infrasound) data provided with the better diagnostic results with several typical learning models.
3. The two reasons shown above encourage the researchers in the heart disease diagnosis using heart sounds and Deep Learning models.

We believe that what Reviewer 1 request us to do could be done through a lot of long-term research works that cannot and need not be done by our work in the manuscript.

2) **Reviewer’s comment:** Additionally, the authors should consider conducting post-analysis on their dataset, especially in terms of evaluating the Signal-to-Noise Ratio (SNR) of the samples. This is important because they are exploring new spectral low-frequency features of heart sounds, which may or may not be as noisy as audible sounds. please check this reference:

Barnawi, Ahmed, Mehrez Boulares, and Rim Somai. 2023. "Simple and Powerful PCG Classification Method Based on Selection and Transfer Learning for Precision Medicine Application" Bioengineering 10, no. 3: 294.

<https://doi.org/10.3390/bioengineering10030294>

**Author’s response**:

We deeply agree that the SNR of the true heart sound data should be checked if any noise but the heart sounds should be excluded. We have checked this already by we did not present it in the manuscript. However, as Reviewer 1 recommended, we had better show the results in the revised manuscript. Please see Line 129 and Figure 2 in the revised manuscript.

**Revised Manuscript**:

Figure 2 presents the FFT results of the signals and noise measured using the experimental setup constructed in this study. It demonstrates that heart sounds caused by cardiac activity can be detected from 1 Hz above. The Signal-to-Noise Ratio (SNR) of these heart sound signals is 33.05 dB. (LINE 129 – 132)

3) **Reviewer’s comment:** the authors should revisit the preprocessing phase, specifically the conversion of sound into images, and ensure that Short-Time Fourier Transform (STFT) and Continuous Wavelet Transform (CWT) do not compromise the low-frequency components during this conversion. We have doubts about this because of two reasons: firstly, Figure 2 shows that components below 1Hz appear flat, and secondly, these conversions are intended to mimic the human perception of audible sound via aggressive filtering of irrelavant sound componants.

**Author’s response**:

We have ensured both the CWT or the STFT would not compromise the low-frequency components at all. The flat frequency contents below 1 Hz in Figure 2 of the original manuscript are shown to come from the internal noise of the instruments used in the experiments. This point can be clearly checked in Figure 2 of the revised manuscript.

Moreover, we don’t agree the last claim of Reviewer 1 because the CWT or STFT is not such a thing. The Fourier and the Continuous Wavelet transforms are the mathematical transform from a function of time to another one of frequency. The relationships between the original functions and the transformed ones may be that the transformed ones would be harmonic decompositions of the original ones. Of course, their digital versions, such as FFTs, may change the relationship more complicated but the validity of the ideal relationship can be guaranteed if proper values of time intervals and the total periods of signal time series data are selected. Please see the textbooks, The Fast Fourier Transform and Its Applications [19,20] for the Fourier transform and Fundamentals of Wavelets [21] for the Continuous Wavelet transform.

[19] Brigham, E. O. (1988). The Fast Fourier Transform (FFT). In The Fast Fourier Transform and Its Applications (Chapter 9). Prentice Hall.

[20] Brigham, E. O. (1988). FFT Transform Applications. In The Fast Fourier Transform and Its Applications (Chapter 10). Prentice Hall.

[21] Goswami, J.C. and Chan, A.K. (2011). Time-Frequency Analysis. In Fundamentals of Wavelets (eds K. Chang, J.C. Goswami and A.K. Chan).

<https://doi.org/10.1002/9780470926994.ch4>

**Reviewer #2 :**

**<Review Comments>**

1) **Reviewer’s comment:** The rationale behind selecting STFT and CWT for data transformation remains undocumented. It would be beneficial to elaborate on the choice of these two transforms.

**Author’s response**:

In this study, we elaborate on the reasons for using Short-Time Fourier Transform (STFT) and Continuous Wavelet Transform (CWT) for data transformation. The primary reason for choosing CWT is its capability for multi-resolution analysis, allowing for maintaining consistent resolution in both infrasound and audible frequency ranges. CWT employs logarithmic frequency resolution in octaves, enabling the transformation of low and regular frequencies into images within an equivalent domain. This is particularly beneficial for maintaining high resolution in the low-frequency domain and effectively separating signal from high low-frequency noise. On the other hand, STFT uses a fixed resolution and is typically set for time-frequency resolutions suited for the audible frequency band. This presents challenges in fitting low-frequency frequency resolution adequately.

based on these technical considerations, CWT was selected as the primary method of data transformation in this study, and this rationale has been added to the research methodology section.

**Revised Manuscript:**

STFT, due to its fixed time-frequency resolution, causes interference in the low-frequency band due to low frequency resolution when the window is adjusted for the audible frequency band. This distortion of the transformation results can lead to significant errors in the assessment of the impact of low-frequency heart diseases, which is a key focus of this study. In contrast, CWT allows for high-frequency resolution in low frequencies through multi-resolution, preventing distortion in the transformation results of the low-frequency range. [22] (LINE 148 - 154)

[22] Graps, A. (1995). An introduction to wavelets. IEEE computational science and engineering, 2(2), 50-61.

2) **Reviewer’s comment:** Information regarding the classification classes isn't explicitly stated. This includes the number of classes and their names, which should be clarified.

**Author’s response**:

Recognizing the importance of providing clear information about classification classes, we have enhanced the content in the Results section. The dataset collected in this study was gathered from various groups of subjects, including patients with different diseases, patients with varying intensities of a particular disease, and post-operative patients.

However, for the classification task in this study, we adopted a simplified approach to reduce the complexity of the dataset and enhance the clarity of the research. Specifically, the classification system was set dichotomously, consisting of two main classes: 'Pre-operative subjects' (Positive class) and 'Normal subjects' (Negative class). Data from post-operative patients were excluded from this classification task. We believe this approach helps focus on the core objectives of our study and makes the interpretation of results clearer.

**Revised Manuscript:**

The classification classes were defined as binary classes, where the 'Positive' group was designated from the dataset labeled as 'Abnormal (Pre-op)', and the 'Negative' group was established from the dataset comprising 'Normal subjects'. (Line 293 - 296)

3) **Reviewer’s comment:** Details about the dataset used for training, specifically the quantity of samples and the data split (e.g., 70% for training and 30% for testing), are missing.

**Author’s response**:

In this study, instead of the standard 7:3 (70% training, 30% testing) data split, we utilized 5-fold cross-validation to divide the data into 80% for training and 20% for validation. The primary reason for this decision was our assessment that the size of the dataset we collected was not yet sufficiently large.

Particularly in cases where the dataset is small, as with ours, the conventional 7:3 split can lead to significant variations in model performance evaluation, depending on the selection of the test set. As observed in the results of our paper, the performance variability when using our dataset was much more significant compared to the larger PhysioNet dataset. This variability can be several times greater depending on the size of the dataset.

The 5-fold cross-validation method was chosen in consideration of these dataset limitations. This approach allows for a more stable evaluation of the model's learning performance and helps reduce the risk of overfitting, especially in small datasets. [27] This decision was made after consulting relevant studies and plays a crucial role in enhancing the reliability of our research results.

**Revised Manuscript:**

To evaluate the model, the cross-validation method [27] was used, and 5-fold cross-validation was performed in the experiment. (Line 188 – 190)

[27] Goodfellow, I., Bengio, Y., & Courville, A. (2016). Machine Learning Basics. In Deep Learning (pp. 121-123). MIT Press.

4) **Reviewer’s comment:** Utilizing the extensive PhysioNet dataset (with 18 classes) raises questions about how many classes were actually chosen for validation

**Author’s response**:

In our study, we utilized the 2016 PhysioNet/CinC Challenge dataset, which primarily offers two dichotomous classes: 'Normal' and 'Abnormal'. The focus of our study is on distinguishing between these two classes, and as such, all analyses and modeling were conducted based on these two categories.

Consequently, we carried out data labeling and one-hot encoding processes centered on these two classes. This approach aligns with the objective of our study, which is to differentiate between normal and abnormal heart sounds, thereby enhancing the clarity of results and ease of interpretation. Therefore, among the various classes provided by the PhysioNet dataset, only these two classes were utilized in our study.

**Revised Manuscript:**

The 2016 PhysioNet/CinC dataset is a valuable resource for developing and evaluating heart sound diagnosis models.[16] It contains a large number of heart sound recordings, which were obtained from different sources, including public databases and hospitals; hence, it provides a diverse set of data to train and test models with binary classes (Normal, Abnormal). (Line 200 - 204)

5) **Reviewer’s comment:** The process for parameter selection in each layer of the model should be explained to provide a clearer understanding

**Author’s response**:

The model used in our study was inspired by the structure of the VGG model, adopting key parameters of the VGG model such as 'same padding', 'feature size doubling', and '1/2 max-pooling'.

However, the major differences from the VGG model lie in the depth of each convolutional layer, the size of the base feature, and the number of features in the dense layers. The selection of these parameters was primarily influenced by the limitations of RAM and VRAM in our team's computing environment. Generally, an increased number of features can improve performance, but it also requires more memory due to the higher number of parameters, leading to hardware constraints.

Therefore, in our study, we chose the optimal values that our computing environment could accommodate for designing our model. We then validated this custom-designed model using the PhysioNet dataset, comparing its performance with existing studies to ascertain if our model demonstrates similar effectiveness. This validation process played a crucial role in verifying the model's feasibility and enhancing the reliability of our research findings.

**Revised Manuscript:**

The foundational design of the CNN model was developed referencing the VGG16 model [24]. Accordingly, layer parameters were set, including the use of same padding and doubling the feature size with increasing depth. Additional parameters, such as the base feature parameter of the first layer and the depth of model, were configured based on the performance capabilities of the computing environment utilized. (LINE 169 – 174)

The computer environment employed comprised a CPU AMD Ryzen 1900X, RAM of DDR3 1333MHz 128 GB, and a GPU NVDIA GEFORCE GTX 1080 Ti (LINE 191 - 193)

[24] Simonyan, K., & Zisserman, A. (2014). Very deep convolutional networks for large-scale image recognition. *arXiv preprint arXiv:1409.1556*.

6) **Reviewer’s comment:** Elucidate the advantages of using LSTM over employing a softmax classifier or feature extraction followed by a traditional machine learning classifier.

**Author’s response**:

We'll clearly explain the primary reasons for using Long Short-Term Memory (LSTM) in our study. Firstly, it's important to note that a softmax classifier was also employed in our CNN-LSTM model. However, there is a significant difference between the approach of traditional machine learning classifiers or feature extraction in Dense layers and the approach of LSTM.

Dense layers primarily extract local features, which reflect fixed patterns in images or time-series data. In contrast, LSTM layers focus on analyzing sequential characteristics such as the continuity or repetitiveness of these local features.[26] Heart sound data inherently possesses characteristics reflecting the regular heartbeat patterns, and these regularities are crucial for identifying abnormal heart sound patterns. LSTM is adept at effectively analyzing and modeling the temporal characteristics and regularities of such sequence data. This is particularly effective for data like heart sounds where temporal continuity is significant, and we believe it is one of the reasons why our CNN-LSTM model achieved high accuracy with fewer parameters. This method of analysis is evidenced in the results of our study, demonstrating that the use of LSTM in processing heart sound data offers superior performance compared to traditional machine learning methods.

**Revised Manuscript:**

In healthy individuals, heartbeats are typically regular, but in cases of illness, irregularities and noise can increase. The classification layer using LSTM can analyze regularities from such sequential data and discern features associated with irregularities. [26] By experimenting with CNN-LSTM, it is possible to preliminarily assess whether there are sequential features even in low-frequency components. (LINE 178 - 183)

[26] Mutegeki, R., & Han, D. S. (2020). A CNN-LSTM approach to human activity recognition. In *2020 international conference on artificial intelligence in information and communication (ICAIIC)* (pp. 362-366). IEEE.

7) **Reviewer’s comment:** Further elaboration on the results would be beneficial to enhance clarity and provide a deeper analysis.

**Author’s response**:

In the original manuscript, our analysis primarily focused on the differences between accuracy and sensitivity. However, in response to the comments received, we have now included additional analyses. We have added detailed examinations of the size of variation within the dataset, and a comparative analysis between the CNN-LSTM and CNN models.

Examining the size of variation within the dataset aids in assessing the model's generalization capability and stability. Finally, the comparative analysis between the CNN-LSTM and CNN models allows for a deeper understanding of the strengths and weaknesses of each model and their suitability for specific datasets.

These additional analyses will enhance the understanding of our research results and add depth to the interpretation of these results. Moreover, they will play a crucial role in enabling other researchers to more easily apply and interpret our findings.

**Revised Manuscript:**

CNN and CNN-LSTM models show a higher standard deviation in performance on the audible dataset, with an average of 2.76%, and on the infrasound dataset, with an average of 2.91%, compared to a lower standard deviation of 0.48% and 0.66% when trained with the PhysioNet dataset. A higher standard deviation in cross-validation suggests a greater tendency towards overfitting, indicating that the dataset size collected through this experiment may not be sufficient. Additionally, the standard deviation of sensitivity is consistently higher than that of specificity, which could be attributed to the dataset ratio, where normal data outnumber abnormal data by a factor of 1.41. CNN-LSTM showed an overall improvement in the average value and standard deviation of Specificity compared to CNN-Dense. However, for Sensitivity, there was a decrease in the average value and a maintenance of the standard deviation in the Audible dataset, while in the Infrasound dataset, the average value increased but with a larger standard deviation. This analysis suggests that while LSTM's sequential feature made it easier to identify regular normal patterns, it appears to have had little impact on the diagnosis of heart sounds in abnormal subjects. (LINE 308 – 323)

8) **Reviewer’s comment:** Considerations about dealing directly with signals rather than transforming them into images should be addressed.

**Author’s response**:

The choice between directly processing the signal and converting it into an image is a significant consideration. In our study, we opted to convert the signal into an image for analysis. This decision was based on the methodology of the current research and the resources available to us.

However, as pointed out in the comments, we have not completely dismissed the possibility of using a raw signal approach. Indeed, we recognize that using raw signals might lead to a decrease in the weight of signals in the audible range due to the size difference in components between the audible and infrasound ranges. This consideration will be addressed in future research.

We also note the existence of previous studies that achieved high diagnostic accuracy using Segmentation and LSTM. These studies segmented the heart sound signals into individual beats and analyzed them using LSTM models. We see this method as potentially applicable to our research and plan to experiment with it if we successfully implement a beat separation algorithm.

These additional considerations regarding data processing methods will be included in the future work section of our study's conclusion. This will contribute to the diversity and depth of our research findings.

**Revised Manuscript:**

Further research could involve benchmarking a variety of models. While different preprocessing techniques or models, such as those other than the currently used CWT, CNN-Dense, or CNN-LSTM, may exhibit higher performance. A deeper understanding can be obtained by testing various advanced models after further expanding the dataset. (LINE 346 – 349)

9) **Reviewer’s comment:** Address the comparison of your results with existing studies where your achieved performance might not be the highest, providing context and potential reasons for variations.

**Author’s response**:

The analysis of the performance differences and comparisons with existing studies is a crucial aspect of our research. One of the main reasons for the somewhat lower performance observed in our study is the size of the dataset. Currently, the dataset we possess is relatively small, which can impact the model's performance.

Additionally, at present, we have not secured sufficient funding for the expansion of our dataset. We plan to expand the dataset and subsequently improve the model's performance once the necessary funding is obtained. We agree that fine-tuning hyperparameters and adjusting learning seeds can incrementally improve current performance.

However, the core argument of our study is not merely about improving individual learning performance but rather emphasizes the impact of frequency component differences in various datasets on learning performance. This highlights the originality and significance of our research and is a critical element that showcases its potential. From this perspective, our study presents a new understanding and approach to specific datasets, which holds significant value beyond just performance differences compared to existing studies.

**Revised Manuscript:**

The training performance of the CNN-Dense and CNN-LSTM models used in our study exhibited accuracies of 92.65% and 93.16%, respectively, which are sufficient for performance validation purposes. While these results are higher than those of the CNN model reported in 2019[10], they are lower compared to the CNN-LSTM[12] model, which achieved a training performance of 98.34%. The significant difference in this model was attributed to an improvement of 2% through the use of dropout compared to no dropout, and the use of a feature size in the LSTM layer that was 5.3 times greater than that in our model. Additionally, there was a notable inclusion of one more Dense layer before the final softmax layer. Although our study assumed that two LSTM layers would be sufficient for extracting sequential features and functioning effectively as a classification layer, the lower performance compared to LSTM-Dense suggests that LSTM might be less effective for classification compared to Dense layers. (LINE 279 – 290)

10) **Reviewer’s comment:** Regarding your designed circuits, clarifications regarding novelty and their compatibility with existing microphones would be beneficial for understanding their applicability.

**Author’s response**:

We recognize the need for a clear explanation of the innovativeness of the circuit designed in our study and its compatibility with existing microphones. Traditional low-frequency responsive microphones have faced constraints in terms of size and cost, and our research has been directed towards overcoming these limitations.

The experimental setup in our study was designed with compatibility with existing microphones in mind. Our developed setup can be applied to microphones using a 1/2 inch external packaging and is designed to be compatible with 1/4 inch and 1 inch microphones through the use of dual adapters. This compatibility with various sizes of microphones indicates that our circuit design has a broader application range compared to existing technologies.

Furthermore, the development of small low-frequency microphones using MEMS (Micro-Electro-Mechanical Systems) technology is one of the focal points of our lab's research. This development of compact microphones can facilitate the miniaturization and cost reduction of equipment for low-frequency signal measurements and, when combined with our study, can expand the possibilities for applications.

**Revised Manuscript:**

This acoustic adapter features a sub-adapter, which can be interchanged depending on the size of the microphone mounted on it. This design allows compatibility not only with the 1/2 inch B&K 4193 microphone but also with smaller MEMS microphones and microphones up to 1 inch in size. (LINE 99 – 102)

Through the experimental equipment, which exhibits flat frequency characteristics in infrasound, the potential of low-frequency heart sounds was confirmed. However, there remains a significant limitation due to the considerable size of the experimental equipment. This is based on a problem that cannot be addressed with conventional small microphones, indicating a need for the development of new ultra-compact infrasound acoustic sensors. (LINE 350-355)

**Associator Editor:**

1. Please ensure that your manuscript meets PLOS ONE's style requirements, including those for file naming. The PLOS ONE style templates can be found at

https://journals.plos.org/plosone/s/file?id=wjVg/PLOSOne_formatting_sample_main_body.pdf and

https://journals.plos.org/plosone/s/file?id=ba62/PLOSOne_formatting_sample_title_authors_affiliations.pdf.

2. Please note that PLOS ONE has specific guidelines on code sharing for submissions in which author-generated code underpins the findings in the manuscript. In these cases, all author-generated code must be made available without restrictions upon publication of the work. Please review our guidelines at https://journals.plos.org/plosone/s/materials-and-software-sharing#loc-sharing-code and ensure that your code is shared in a way that follows best practice and facilitates reproducibility and reuse.

3, Thank you for stating in your Funding Statement:

‘This study was supported by the Korea-led K-Sensor Technology Development Program for Market Leadership (RS-2022-00154770, Next-generation piezoelectric based motion sensor platform) funded By the Ministry of Trade, Industry \& Energy(MOTIE, Korea). The funders had no role in study design, data collection and analysis, decision to publish, or preparation of the manuscript.”

Please provide an amended statement that declares *all* the funding or sources of support (whether external or internal to your organization) received during this study, as detailed online in our guide for authors at http://journals.plos.org/plosone/s/submit-now. Please also include the statement “There was no additional external funding received for this study.” in your updated Funding Statement.

Please include your amended Funding Statement within your cover letter. We will change the online submission form on your behalf.

**Revised Funding Statement:**

This study was supported by the 2017 Seoul St. Mary’s Hospital Leading Specialization Project (KC17TESI0684, Development of the optimized sensor and the deep learning – based automated cardiac sound analysis system for digital stethoscope) funded By Seoul St. Mary’s Hospital
