# Supplementary material for: Effects of precise cardio sounds on the success rate of phonocardiography
Source: PLoS One. 2024 Jul 15;19(7):e0305404. doi: 10.1371/journal.pone.0305404 (PMC11249217; doi:10.1371/journal.pone.0305404)
Supplement: Attachment — Submitted filename: Response to reviewers 2.docx [file pone.0305404.s005.docx]

**Responses to Reviewer’s Comments**

February 23, 2024

Dear reviewer in *PLOS ONE*

**Reviewer #1:**

***<Review Comments>***

1) **Reviewer’s comment:** the authors didn't respond to the first comment that they need to establish some clinical relevance to infrasound and cardiac diagnosis.

**First comments in the previous review:** *In this paper, the authors investigate the impact of low-frequency components, referred to as 'infrasound,' on the accuracy of classifying PCG (Phonocardiogram) sounds. The approach is novel and intriguing, though the authors need to establish, theoretically or clinically, whether infrasound components are directly related to the diagnosis of medical conditions. Nevertheless, the study provides valuable insights, suggesting that exploring this avenue is worthwhile*.

**Author’s response:**

We regret we did not provide our specific answer to the first comment of Reviewer 1. However, due to the last sentence of his/her comments given above, we thought we don’t have to respond to the comments specifically. Moreover, since our answers to the second comments of Reviewer 1 includes our answers to the first comments implicitly, we have missed the step required by the reviewer.

As is requested in the first sentence of the current comments from Reviewer 1, our specific responses to the first comments would be as follows:

As in our previous responses, we believe that we have already ‘theoretically’ and experimentally established whether infrasound components are directly related to the heart conditions in the previous first revision. It is shown that the detected sounds including the infra-frequency components are from the heart, (Our experimental procedures for acquisition of heart sounds would prove it.) hence, they could be highly related to the physical conditions of heart. This shows the relationship between heart sounds including infra-frequency components and the heart condition. Since Reviewer 1 requests to establish, ‘theoretically or clinically’, we have almost done his/her request already.

The remaining may be to establish the relationship between the infra sounds and the diagnosis of medical conditions. Obviously, it cannot be done theoretically. Hence, we can do it clinically. In order to show the clinical relationship between the heart sounds including infra sounds and the medical conditions, we need to check the relationship between those, which have been done in our study presented in the manuscript. It is, justifiably, not complete, but it shows the high probability. So, we conclude that more intensive studies are required. We have claimed almost identical claims in our previous responses to the comments from Reviewer 1.

Even though the complete relationship is not established in our study, we have shown high possibility of the close relationship. The complete one would be the final goal of the researchers in this research field. Every researcher would do something as one step to the final goal and we did one. Therefore, we believe, it is not appropriate to claim that we need to establish, theoretically or clinically, whether infrasound components are directly related to the diagnosis of medical conditions. This point was claimed in our answers to the previous responses for the first revision.

2) **Reviewer’s comment:** Also the response to the other comments were unsatisfactory.

**Author’s response:**

We are sorry that Reviewer 1 is not happy with our responses to his/her comments. However, we don’t see any scientific reason of his/her complaints. Therefore, we are receiving this sentence as an expression of his/her personal, emotional feeling, which we do not need to soothe.
