# Supplementary material for: Effects of precise cardio sounds on the success rate of phonocardiography
Source: PLoS One. 2024 Jul 15;19(7):e0305404. doi: 10.1371/journal.pone.0305404 (PMC11249217; doi:10.1371/journal.pone.0305404)
Supplement: Attachment — Submitted filename: Response to reviewers 3.docx [file pone.0305404.s007.docx]

**Responses to Reviewer’s Comments**

May 16, 2024

Dear editor and reviewer in *PLOS ONE*

We are profoundly grateful for the insightful critiques and constructive suggestions provided on our manuscript titled **“Effects of precise cardio sounds on the success rate of phonocardiography”**, control number PONE-D-23-23289.

In the revised version, we have highlighted all changes for your convenience; these are underlined and colored in blue. This is to ensure that our revisions are easily identifiable and can be efficiently evaluated.

**To Editor:**

Upon reviewing the references, it was identified that the previously cited references 5 and 9 were the same. Consequently, reference 9 has been amended to reference 5 in the revised document.

**Revised manuscript:**

Heart sounds provided in PhysioNet can be successfully classified into two categories (normal and abnormal) with a 98.3% success rate by using long short-term memory (LSTM) and segmentation based on logistic-regression hidden semi-Markov model (HSMM)-based techniques. [5]

**Reviewer #3:**

***<Review Comments>***

1) **Reviewer’s comment:** It would be more appropriate for the authors to define abbreviations upon first appearance in the main text such as micro-electromechanical systems (MEMS).

**Author’s response:**

We appreciate your attention to detail regarding the use of abbreviations in our manuscript. Upon your suggestion, we have revised the text to include a definition for MEMS at its first occurrence as follows: MEMS (Micro-Electromechanical Systems). Additionally, we conducted a thorough review of the document to ensure that all other necessary abbreviations are similarly defined at their first mention, thereby improving the readability and accessibility of our paper.

**Revised manuscript:**

This design allows compatibility with the ½-inch B&K 4193 microphone and also with miniature MEMS(Micro-Electromechanical Systems) microphones and large microphones up to 1 inch in size.(Line Number 91 – 93)

2) **Reviewer’s comment:** The authors have inserted Eq. 2 and 3 in the manuscript without citing them properly inside the manuscript. Kindly check it and perform the required amendment.

**Author’s response:**

We appreciate your observation regarding the lack of proper citation for Equations 2 and 3. To address this issue, we have revised the relevant sections to include and properly cite these equations, particularly in our explanation of the Continuous Wavelet Transform (CWT). This amendment ensures that all mathematical expressions are accurately referenced within the text, enhancing both the credibility and scholarly integrity of our manuscript. Thank you for bringing this to our attention.

**Revised manuscript:**

Therefore, we used the continuous wavelet transform (CWT), which is defined as the integral (Eq. 2) over all time of the product of the signal f(t) and a scaled and translated version of the mother wavelet ψ. Here, ψb,a(t) represents the mother wavelet (Eq. 3) modified by the scale parameter a and translation parameter b, where a influences the dilation of the wavelet, allowing it to capture features at various frequencies, and b adjusts the wavelet’s position, facilitating the analysis of the signal’s temporal characteristics. [20] (Line Number 130 – 136)

3) **Reviewer’s comment:** It would be more appropriate for the authors to define abbreviations of MATLAB as a proprietary multi-paradigm programming language and numeric computing environment developed by MathWorks. This will contribute more knowledge to the reader.

**Author’s response:**

We appreciate your suggestion to provide a more detailed explanation of MATLAB in our manuscript. In response to your feedback, we have included a comprehensive definition of MATLAB as follows: MATLAB, a proprietary multi-paradigm programming language and numerical computing environment, is developed and distributed by MathWorks. This enhancement aims to enrich the reader's understanding of the context in which MATLAB is utilized within our study.

**Revised manuscript:**

In the Wavelet Toolbox of MATLAB, a proprietary multi-paradigm programming language and numerical computing environment developed and distributed by MathWorks, the CWT function is utilized to perform the wavelet transform. (Line Number 146 – 148)

4) **Reviewer’s comment:** A minor inconsistency in the usage of the term "CWT” was noticed. In some sentences, it was written in capital letters, while in others, it was written in lowercase. To maintain consistency and clarity throughout the text, I suggest standardizing the writing of "cwt" by either capitalizing it or using lowercase letters consistently.

**Author’s response:**

Thank you for pointing out the inconsistency in the use of the abbreviation "CWT." To address this, we have standardized the term to "CWT" throughout the manuscript when referring to the concept of Continuous Wavelet Transform. Additionally, regarding the term "dense" as it relates to TensorFlow's layers, we have corrected all instances to use lowercase "dense" consistently throughout the document. This distinction clarifies the text and adheres to the proper usage of technical terms. Your feedback has significantly improved the manuscript’s consistency and clarity.

**Revised manuscript:**

1. Therefore, we used the continuous wavelet transform (CWT), which is an algorithm that transforms a signal in the time domain to the time–frequency domain by translating and scaling a basis function called a wavelet. (Line Number 130 – 132)
2. In contrast, CWT allows for high-frequency resolution at low frequencies by multi-resolution , preventing distortion in the transformation results of the low-frequency range. (Line Number 143 – 145)
3. In the Wavelet Toolbox of MATLAB, a proprietary multi-paradigm programming language and numerical computing environment developed and distributed by MathWorks, the CWT function is utilized to perform the wavelet transform. The CWT function was implemented in a frequency range of 2–300 Hz, using a Morlet wavelet as the basis wavelet. (Line Number 148 – 150)
4. STFT and CWT for the heart-sound signal were obtained from the experimental equipment. Compared to the STFT image (Fig. 3A), the CWT image (Fig. 3B) provides a clearer representation of the frequency characteristics of the signal and enables feature extraction in both low- and high-frequency bands. (Line Number 153 – 156)
5. Different preprocessing techniques or models, such as those other than the currently-used CWT, CNN-dense, or CNN-LSTM, may achieve higher accuracy than those that do use them. (Line Number 333 – 335)
5) **Reviewer’s comment:** Moderate English grammar editing is required throughout the manuscript, for example: a.In the section of train results, “However, for Sensitivity, there was a decrease in …). moderate editing is required.

**Author’s response:**

We acknowledge your concerns regarding the grammatical accuracy in our manuscript, particularly in the sections added during the previous revision. We have taken your feedback seriously and have resubmitted the document to a professional editing service for thorough proofreading and correction. This ensures that the text now meets the high standard of clarity and grammaticality expected in scholarly communication.
