# Supplementary material for: Effects of precise cardio sounds on the success rate of phonocardiography
Source: PLoS One. 2024 Jul 15;19(7):e0305404. doi: 10.1371/journal.pone.0305404 (PMC11249217; doi:10.1371/journal.pone.0305404)
Supplement: Attachment — Submitted filename: reviewer comments - Mai-08042024.docx [file pone.0305404.s006.docx]

**Reviewer Comments**

**Dear Editor(s), April 08, 2024**

I am pleased to submit my comments for the submitted manuscript (**PONE-D-23-23289R2)** entitled “**Effects of precise cardio sounds on the success rate of phonocardiography”** to be taken into consideration for publication by your esteemed journal “***PLOS ONE”***.

Heart sounds, primarily caused by blood pumping, offer crucial insights into cardiovascular health, including muscle status and blood flow. Auscultation, a simple technique not requiring complex equipment, utilizes these sounds for heart condition diagnosis. The difficulties in auscultation may be easily illustrated by the fact that even good cardiology fellows could diagnose heart diseases with an accuracy of only 56.2%. Recent research explores artificial intelligence for automatic diagnosis via heart sounds, mostly focusing on improving learning models and datasets. However, current capacitive MEMS microphones, commonly used for heart sound capture, have limitations in detecting signals below 20 Hz, despite human hearts producing low-frequency sounds. This study investigates whether including these low-frequency components, not typically used in diagnosis, could enhance diagnostic accuracy. Due to the unavailability of commercial microphones for low-frequency heart sounds, an experimental setup was created for measurement.

In this study, the authors have successfully investigated the effects of infrasound inclusion in heart sound data analysis. The authors established a heart sound measurement environment that comprised a microphone, amplifier, and data acquisition (DAQ) equipment. By standardizing the measurement method, the authors aimed to obtain a reliable dataset that could be used for accurate heart sound diagnosis. The Continuous Wavelet Transform was used to analyze changing frequency characteristics over time and distribute frequency resolution appropriately between the low-frequency and audible frequency bands. For the learning model, a Convolutional Neural Network was used, a basic model optimized for image classification, and a CNN-LSTM that added sequential feature extraction. The performance of the learning model was validated using the PhysioNet 2016 CinC dataset, and we used our collected dataset to confirm the difference in diagnostic performance depending on the presence or absence of low-frequency components. Incorporating low-frequency component data into the dataset improved the deep-learning model’s performance, with increase in accuracy and sensitivity by 2% and 4% respectively.

The authors have done good work on the title “**Effects of precise cardio sounds on the success rate of phonocardiography**”. It will add new knowledge and new areas of research to the subject area compared with other published material.

**However, i have some minor concerns:**

1. It would be more appropriate for the authors to define abbreviations upon first appearance in the main text such as micro-electromechanical systems (MEMS).
2. The authors have inserted Eq. 2 and 3 in the manuscript without citing them properly inside the manuscript. Kindly check it and perform the required amendment.
3. It would be more appropriate for the authors to define abbreviations of MATLAB as a proprietary multi-paradigm programming language and numeric computing environment developed by MathWorks. This will contribute more knowledge to the reader.
4. A minor inconsistency in the usage of the term "CWT” was noticed. In some sentences, it was written in capital letters, while in others, it was written in lowercase. To maintain consistency and clarity throughout the text, I suggest standardizing the writing of "cwt" by either capitalizing it or using lowercase letters consistently.
5. **Moderate English grammar editing is required throughout the manuscript, for example:**
a. In the section of train results, “However, for Sensitivity, there was a decrease in …). moderate editing is required.

Best regards,

Dr. Mai Abdel Haleem Abusalah

Faculty of Medical Allied Science,

Al-Ahliyya Amman University,

Amman, Jordan.

Tel: +962-796862347

e-mail: [ellamomo88@yahoo.com](mailto:ellamomo88@yahoo.com)
